# Reference intervals of complete blood count parameters for individuals aged 80 to 89 years in Guizhou, China: A STROBE-compliant retrospective study

**DOI:** 10.1097/MD.0000000000030859

**Published:** 2022-10-07

**Authors:** Jinlie Long, Xiuhong Wang, Jianbo Yuan, Jianru Yang, Jie Zhou, Yuan Chen, Enxi Hu, Yuanzhong Zhou, Xun Min

**Affiliations:** a Department of Laboratory Medicine, Affiliated Hospital of Zunyi Medical University, Zunyi, Guizhou, China; b School of Laboratory Medicine, Zunyi Medical University, Zunyi, Guizhou, China; c Department of Orthodontics, Affiliated Stomatological Hospital, Zunyi Medical University, Zunyi, Guizhou, China; d School of Public Health, Zunyi Medical University, Zunyi, Guizhou, China.

**Keywords:** aged, blood cell count, reference intervals

## Abstract

The reference intervals of complete blood count (CBC) parameters were commonly based on healthy individuals aged 20 to 79 years. However, these values are not optimal for correct clinical diagnosis in older individuals (e.g., 80–89 years). Although the reference intervals for this age group have been reported in China, there is no population-based report in Guizhou province. A total of 481 healthy adults (238 males and 243 females) aged 80 to 89 years were recruited from Affiliated Hospital of Zunyi Medical University in Guizhou. The CBC parameters were detected by Sysmex XN-9000 automatic hematology analyzer. The reference intervals of the components were analyzed according to the guidelines of International Federation of Clinical Chemistry. This study reported the reference intervals of CBC parameters. There were significant differences were examined in some reference intervals between the different gender groups, especially for RBC-related parameters. Compared with national standards, the most of all conventional reference intervals for CBC parameters were decreased. The present study provided the local reference intervals of CBC parameters for individuals aged 80 to 89 years in Guizhou, China. Some of our results were sex-specific, and most of our results show lower values while comparing with commonly used reference intervals in China. Therefore, more attentions should be paid to these differences, and accurate reference intervals will facilitate clinical diagnosis and decision-making in these populations.

## 1. Introduction

The reference interval, first mentioned in the 1960s by Grsbeck et al, has a distribution of values ranging from 2.5% to 97.5%.^[1]^ Appropriate reference interval is beneficial to clinical diagnosis and treatment.^[2,3]^

As we known, the complete blood count (CBC) parameters including red blood cell (RBC)-, white blood cell (WBC)-, and platelet (PLT)-related parameters. For example, in the CBC parameters of Sysmex XN-9000 automatic hematology analyzer, the RBC-related parameters were consist of RBC count, hemoglobin (Hb), hematocrit (Hct), mean corpuscular volume (MCV), mean corpuscular hemoglobin (MCH), MCH concentration (MCHC), and RBC distribution width (RDW); The WBC-related parameters contained WBC count, neutrophil (NEUT) count/fraction, lymphocyte (LYMPH) count/fraction, monocyte (MONO) count/fraction, eosinophil (EO) count/fraction, and basophil (BASO) count/fraction; PLT-related parameters including PLT count, PLT distribution width (PDW), mean PLT volume (MPV), PLT-larger cell ratio (P_LCR), and plateletcrit (PCT). The reference intervals for CBC parameters, commonly used in disease diagnosis and health screening. For example, in RBC index, previous study has shown that lower blood Hb levels were associated with poor cognitive function and Alzheimer disease.^[4]^ And higher MCV was independently positively associated with post-stroke depression.^[5]^ Among WBC-related parameters, increased WBC counts correlate with severity of type 2 diabetes and metabolic syndrome.^[6]^ And the NEUT-LYMPH ratio positively correlates with cardiovascular disease severity and prognosis.^[7]^ For the PLT index, elevated PLT counts were positively associated with insulin resistance,^[8]^ and MPV is strongly associated with hypertension and obesity.^[9]^

The reference intervals of CBC parameters might be influenced by various objective factors, such as population gender. One study showed that most of the CBC parameters in Korean adults were higher in males than in females.^[10]^ Another study indicated that most RBC-related parameters were sex-specific in the healthy Han population.^[11]^ In addition, the influence of region on the above parameters cannot be ignored. The researchers compared the CBC parameters in Shangri La, Yunnan (average altitude of 3280 meters) and Shanghai (average altitude of 4 meters) of China. The results showed that RBC-related parameters in high-altitude areas were higher than those in low-altitude areas, while PLT-related parameters in high-altitude areas were lower than those in low-altitude areas.^[12]^ In addition, the population age may affect CBC parameters. Previous studies have shown that there are differences in the hematopoietic capacity of different age groups. With the increase of age, the hematopoietic function of bone marrow gradually declines.^[13]^

Guizhou is one of the important provinces in the southwest of China. With the rapid development of economy and society, the lifestyle or nutritional status has undergone tremendous change in the past few decades. In addition, the population size and age composition of the district has also changed greatly. According to the data of the seventh national census, as of November 1, 2020, the total population of Guizhou has reached 38.5621 million, and the degree of aging was further deepened than that of sixth census.^[14,15]^ The populations aged 80 to 89 years reached 53,952 in sixth census, thus, the health of this part of the populations cannot be ignored. The CBC values as a routine method for screening the health status of the population in clinical work, however, the reference intervals of these parameters are based on different age group in international and national standards, respectively, for example, the former usually based on populations aged 20 to 50 years,^[16]^ but the latter commonly based on individuals aged 20 to 79 years.^[17]^ Therefore, these values may not be applicable to the individuals aged 80 to 89 years in Guizhou due to the influence of region, age, diet, and other factors, and it is imperative to determine the CBC value of this population in Guizhou. In this study, we recruited 481 healthy adults (238 males and 243 females) between the ages of 80 and 89 from the Affiliated Hospital of Zunyi Medical University in Guizhou. The CBC parameters were detected by Sysmex XN-9000 automated hematology analyzer from January 2018 to December 2020. Data analysis was performed according to the guideline of the Clinical and Laboratory Standards Institute EP28A-3C.^[18]^ This study will provide valuable data support for clinical practice.

## 2. Materials and Methods

### 2.1 Subject selection

This study was conducted at the Affiliated Hospital of Zunyi Medical University in Guizhou province, China. While elderly persons came to above hospital for health check, the volunteer would be included in our study if they met all of the following criteria: 80 to 89 years old; Without acute and chronic diseases, surgery or trauma within 6 months, blood donation or transfusion within 4 months, smoking and drinking alcohol; Resided in Guizhou; Willing to participate in our study. We recruited 498 old adults between January 2018 and December 2020, and missing data (n = 14) were excluded, and the outliers (n = 3) were also excluded. Ultimately, 481 old persons were included in present project. All volunteers signed an informed consent form before enrolling in present study. The protocol of this study was approved by the ethics committee of the Affiliated Hospital of Zunyi Medical University in Guizhou.

### 2.2 Sample collection

After fasting by a professional nurse for at least 8 hours, venous blood (2 mL) of volunteers was drawn into a professional anticoagulant (EDTA-K2) tube (Zhejiang Gongdong Medical Instrument Co., Ltd., China). Then immediately invert the test tube 6 to 8 times and send it to the laboratory for testing within 1 hour.

### 2.3 Quality assurance

A Sysmex XN-9000 automated hematology analyzer (Sysmex Corporation, Japan) was used to measure the 32 routine CBC parameters in line with the manufacturer’s instructions. The precision or accuracy of the hematology analyzer was assessed according to the guideline for International Council for Standardization.^[19,20]^ Quality control was performed by high and low levels before and after measurement.

### 2.4 Statistical analysis

On the basis of Reed et al suggestion,^[21]^ the outliers were identified by the 1/3 rule and the D/R ratio, where the D was the absolute difference between the extreme value and the next maximum (or minimum) value, and R was range of all values. Presenting the reference intervals of CBC parameters separately in each gender group was necessary according to Harris and Boyd test.^[22]^ According to the CLSI EP28A-3C guideline,^[23]^ 90% confidence intervals (CIs) for CBC parameters were established. Mann–Whitney U test was utilized to compare the difference of all parameters between 2 groups after normality test was done by the Kolmogorov−Smirnov. Differences were considered statistically significant once the *P* value was less than .05. Data analysis was carried out by the software of SAS (version 9.4).

## 3. Results

### 3.1 Study population

Four hundred ninety eight old individuals were enrolled between January 2018 and December 2020, the missing data (n = 14) and outliers (n = 3) were excluded. Finally, 481 persons (238 males and 243 females) aged 80 to 89 years were included to determine the reference intervals of CBC parameters.

### 3.2 Reference intervals of RBC-related parameters for healthy adults aged 80 to 89 years

The median, interquartile range (IQR), reference intervals, and 90% CIs of 7 routine RBC-related parameters in separate gender group are showed in Table 1. As can be seen from the table, the values of RBC count, Hb level, Hct, MCH, and MCHC in men were significantly higher than in women (all *P* < .001). However, there was no significant difference in MCV and RDW between different gender groups (*P* > .05). Comparing with the standards of standard reference intervals for blood cell analysis of the People’s Republic of China’s health industry (WS/T 405-2012),^[17]^ all the reference intervals were significantly decreased (*P* < .001) except the Hb level, Hct in women (RDW not given in the document).

**Table 1:** Sex-specific medians, IQRs and reference intervals for red blood cell-related parameters obtained from healthy adults aged 80 to 89 years.

Parameter	Sex	Median	IQR	*P* value	Reference interval	Lower limit with 90% CI	Upper limit with 90% CI	National standard†
RBC, ×10^12^/L	M	4.53	4.15–4.97	<.001	3.32–5.73***	3.26–3.39	5.66–5.79	4.3–5.8
	F	4.26	3.99–4.52		3.40–5.10***	3.35–3.45	5.05–5.14	3.8–5.1
Hb, g/L	M	144	130–154	<.001	107–177	105–109	175–179	130–157
	F	132	123–140		106–156	104–107	154–157	115–150
Hct, L/L	M	0.43	0.40–0.46	<.001	0.33–0.53***	0.32–0.33	0.52–0.53	0.40–0.50
	F	0.40	0.37–0.42		0.33–0.47	0.32–0.33	0.47–0.47	0.35–0.45
MCV, fL	M	94.6	91.6–97.3	.239	81.9–107.4***	81.2–82.6	106.7–108.1	82–100
	F	94.3	90.7–97.0		83.6–104.3***	83.1–84.2	103.7–104.8	
MCH, pg	M	31.4	30.5–32.8	<.001	26.7–36.4***	26.4–26.9	36.1–36.7	27–34
	F	31.0	29.9–31.9		27.0–34.7***	26.8–27.2	34.5–34.9	
MCHC, g/L	M	332	327–339	<.001	313–353***	312–314	352–354	316–354
	F	328	322–334		310–346***	309–311	345–347	
RDW,%	M	13.3	12.9–14.0	.317	11.4–15.7	11.3–11.5	15.6–15.8	-
	F	13.2	12.8–13.9		11.6–15.1	11.6–11.7	15.0–15.2	

CI = confidence interval, F = female, Hb = hemoglobin, Hct = hematocrit, IQR = interquartile range, M = male, MCH = mean corpsular hemoglobin, MCHC = mean corpsular hemoglobin concentration, MCV = mean corpuscular volume, RBC = red blood cell, RDW= RBC distribution width.

*** *P*-value <.001 between our results and the national standard.

† Standard reference intervals for blood cell analysis of the People’s Republic of China’s health industry (WS/T 405-2012).

### 3.3 Reference intervals of WBC-related parameters for healthy adults aged 80 to 89 years

Table 2 shows the median, IQR, reference intervals and 90% CIs for 11 routine WBC-related parameters in 2 different groups. The values of MONO count/fraction and EO count/fraction in males were significantly higher than in females (*P* < .001 for MONO count/fraction, and *P* < .05 for EO count/fraction.). However, there were no significant difference in WBC count, NEUT count/fraction, LYMPH count/fraction, and BASO count/fraction was examined between the male and female group. Compared with WS/T 405-2012 criteria, the WBC count, NEUT count, LYMPH count and fraction, EO count/fraction were significantly lower, while the MONO and BASO count/fraction were significantly higher (*P* < .001). Additionally, the NEUT fraction was comparable to the value of the document (*P* > .05).

**Table 2:** Sex-specific medians, IQRs and reference intervals for white blood cell-related parameters obtained from healthy adults aged 80 to 89 years.

Parameter	Sex	Median	IQR	*P* value	Reference interval	Lower limit with 90% CI	Upper limit with 90% CI	National standard†
WBC, ×10^9^/L	M	6.14	5.09–7.48	.346	2.99–9.83***	2.80–3.17	9.65–10.02	3.5–9.5
	F	6.15	5.10–7.00		3.20–9.22***	3.04–3.36	9.05–9.38	
NEUT, ×10^9^/L	M	3.54	2.89–4.45	.233	1.45–5.94***	1.33–1.57	5.82–6.07	1.8–6.3
	F	3.42	2.77–4.29		1.22–5.98***	1.09–1.34	5.85–6.11	
NEUT, %	M	58	53–64	.825	40.9–74.3	40.0–41.8	73.4–75.2	40–75
	F	58	51–64		38.4–76.5	37.3–39.4	75.4–77.5	
LYMPH, ×10^9^/L	M	1.80	1.40–2.27	.437	0.34–3.57***	0.26–0.43	3.49–3.66	1.1–3.2
	F	1.90	1.47–2.38		0.66–3.24***	0.59–0.73	3.18–3.31	
LYMPH, %	M	30	25–36	.072	13.9–47.1***	13.0–14.8	46.2–48.0	20–50
	F	31	26–38		14.6–49.0***	13.7–15.6	48.1–49.9	
MONO, ×10^9^/L	M	0.52	0.43–0.63	<.001	0.22–0.85***	0.21–0.24	0.83–0.87	0.1–0.6
	F	0.47	0.37–0.57		0.20–0.75***	0.19–0.22	0.74–0.77	
MONO, %	M	8	7–9	<.001	4.6–12.3***	4.4–4.8	12.1–12.5	3–10
	F	7	6–9		3.9–11.7***	3.7–4.1	11.5–11.9	
BASO, ×10^9^/L	M	0.05	0–0.07	.820	0.00–0.11***	0.00–0.00	0.11–0.11	0–0.06
	F	0.05	0–0.07		0.00–0.11***	0.00–0.00	0.10–0.11	
BASO, %	M	1	0–1	.911	0.00–1.63***	0.00–0.00	1.58–1.69	0–1
	F	1	0–1		0.00–1.62***	0.00–0.00	1.57–1.67	
EO, ×10^9^/L	M	0.15	0.09–0.23	.045	0.00–0.54***	0.00–0.00	0.52–0.56	0.02–0.52
	F	0.13	0.07–0. 20		0.00–0.42***	0.00–0.00	0.41–0.44	
EO, %	M	2	1–4	.049	0.00–7.99***	0.00–0.00	7.72–8.26	0.4–8.0
	F	2	1–3		0.00–6.90***	0.00–0.00	6.67–7.14	

BASO = basophil, CI = confidence interval, EO = eosinophil, F = female, IQR = interquartile range, LYMPH = lymphocyte, M = male, MONO = monocyte, NEUT = neutrophil, WBC = white blood cell.

*** *P*-value <.001 between our results and the national standard.

† Standard reference intervals for blood cell analysis of the People’s Republic of China’s health industry (WS/T 405-2012).

### 3.4 Reference intervals of PLT-related parameters for healthy adults aged 80 to 89 years

In Table 3, we present the median, IQR, reference intervals, and 90% CIs of 5 routine PLT-related parameters in male and female groups. We found the values of PLT count and PCT in male were significantly lower than in female (*P* < .001), but there were no significant differences in the values of PDW, MPV, and P_LCR between 2 different gender groups (*P* > .05). Comparing with the standards of WS/T 405-2012, the PLT count was significantly decreased (*P* < .001).

**Table 3:** Sex-specific medians, IQRs and reference intervals for platelet -related parameters obtained from healthy adults aged 80–89 years.

Parameter	Sex	Median	IQR	*P* value	Reference interval	Lower limit with 90% CI	Upper limit with 90% CI	National standard†
PLT, ×10^9^/L	M	172	145–203	<.001	84–267***	79–89	262–272	125–350
	F	196	160–239		88–316***	81–94	309–322	
PDW,%	M	14.0	12.1–16.1	.666	8.4–20.4	8.0–8.7	20.0–20.7	-
	F	13.7	12.1–15.8		8.7–19.7	8.4–9.0	19.4–20.0	
MPV, fL	M	11.2	10.4–12.0	.958	9.0–13.6	8.9–9.1	13.5–13.7	-
	F	11.2	10.5–12.0		9.1–13.4	9.0–9.2	13.3–13.6	
P_LCR	M	34.9	28.2–41.2	.882	17.2–53.5	16.3–18.2	52.5–54.5	-
	F	34.5	28.9–41.0		18.0–52.5	17.0–18.9	51.6–53.5	
PCT	M	0.19	0.17–0.22	<.001	0.10–0.29	0.10–0.11	0.28–0.29	-
	F	0.22	0.18–0.26		0.11–0.34	0.11–0.12	0.33–0.34	

CI = confidence interval, F = female, IQR = interquartile range, M = male, MPV = mean platelet volume, PCT = plateletcrit, PDW = PLT distribution width, P_LCR = PLT-larger cell ratio, PLT, platelet.

*** *P*-value <.001 between our results and the national standard.

† Standard reference intervals for blood cell analysis of the People’s Republic of China’s health industry (WS/T 405-2012).

## 4. Discussion

The present study provided the evidence of the critical gap in 23 routinely reported CBC parameters reference intervals for healthy elderly individuals aged 80 to 89 years in Guizhou, China, which may provide basic evidence support for the diagnosis of diseases. Our data presented that most of all routine reference intervals for CBC parameters were decreased when compared to the standards of WS/T 405-2012, and there were significant differences in CBC parameters between the male and female groups. In the RBC-related parameters, the most values of above-mentioned parameters in males were higher than in females. For WBC-related parameters, only the MONO and EO count/fraction in males were higher than in females. However, as for PLT index, the lower values of PLT count and PCT were checked in males when compared with females.

CBC parameters were often used by physicians for disease screening and diagnosis, which could be used as the biomarkers of anemia,^[24]^ infection risk,^[25]^ cardiovascular diseases,^[26]^ and so on. However, these parameters may be affected by lifestyle, diet, race, etc.^[11]^ Furthermore, the influence of age on the reference intervals of CBC parameters could not be ignored.^[27]^ Therefore, it is necessary to establish local normal reference intervals for CBC parameters in the elderly. In present study, a total of 498 healthy adults aged 80 to 89 years were enrolled, the missing data and outliers were excluded, and 481 individuals (238 males and 243 females) were finally included for establishing reference intervals.

As we known, RBC-related parameters are expected to be used in the prediction of various diseases. The RBC count maybe as an effective indicator of microvascular complications in patients with type 2 diabetes mellitus.^[28]^ For Hb level, it can be used as a clinical biomarker for ocular metastasis in male liver cancer patients.^[29]^ In Hct, this could serve as a predictor of intradialytic morbid events.^[30]^ As for MCV, the higher MCV was positively associated with mortality in patients with chronic kidney diseases.^[31]^ The low MCH, low MCHC, high RDW were positively associated with exacerbation of chronic obstructive pulmonary disease,^[32]^ elevated risks of developing depressive symptoms,^[33]^ increased risk of hemorrhagic transformation among acute ischemic stroke patients,^[34]^ respectively. In present study, we presented the reference intervals for 7 routine RBC-related parameters, including RBC count, Hb level, Hct, MCV, MCH, MCHC, and RDW. The above results except Hb level and Hct in females were lower than the standards of WS/T 405-2012 (the reference value of RDW was not given in the document).^[17]^ This may be due to the progressive decline of hematopoiesis with age in the older participants in our study. Compared with individuals of the same age group in Beijing, China,^[35]^ our results (the values of RBC count, Hb level, and Hct) were higher, probably due to the participants of our study resided in higher altitude area, which was positively correlated with the above values.^[36]^ However, the differences in MCV and MCHC between our study and the aforementioned studies are unclear, possibly due to differences in population or diet. In addition, in present study, the values of RBC-related parameters (except MCH and RDW) in males were higher than in females, which may be due to higher androgen levels in males.^[37]^

Similarly, in recent years, more and more studies have shown that WBC-related parameters were used as indicator of diseases in clinical diagnosis. Higher WBC counts within the normal range were independently associated with sarcopenia.^[38]^ The ratio of NEUT-to-MONO could be used as a potential marker indicating the severity of knee osteoarthritis.^[39]^ In our study, the reference intervals of 11 routine WBC-related parameters were established, including WBC count, NEUT count/fraction, LYMPH count/fraction, MONO count/fraction, BASO count/fraction, and EO count/fraction. Comparing with the standards of WS/T405-2012, the reference intervals of WBC-related parameters (except NEUT fraction, MONO, and BASO count/fraction) in present study were decreased, probably due to the lower hematopoietic capacity of the elderly. Our results (the WBC count, NEUT, and EO fraction) were lower than the previous study in Beijing, China,^[35]^ probably due to different living environment and diet. However, the reason for the differences in MONO and EO count/fraction values between our research and aforementioned studies is unclear.

The PLT-related parameters also played an important role for diagnosis of clinical diseases. The high PLT counts were positively associated with severely periodontitis.^[40]^The PDW has the potential to be a new biomarker for predicting stroke.^[41]^ And the elevated MPV was associated with increased incidence of hypertension.^[42]^ Buch et al found the elevated P-LCR in patients with diabetic retinopathy and diabetic nephropathy compared with patients who have not yet observed the above-mentioned complications.^[43]^ For PCT, it could be used to predict the presence and severity of hyperemesis gravidarum.^[44]^ This study reports reference intervals for 5 routine PLT-related parameters, namely PLT count, PDW, MPV, P_LCR, and PCT. The PLT count in our study was lower than the standards of WS/T405-2012 (only the PLT count was given), which may be due to the lower hematopoietic function of bone marrow in older populations. However, the reasons for the difference in PLT count and PCT between the male and female were still unclear.

One of the strengths of our study was that the healthy participants were based on stringent exclusion criteria, avoiding the influence of confounding factors on our findings as much as possible, thus guaranteeing the reliability of the results; Secondly, the study was conducted in accordance with CLSI guidelines. Therefore, every step of process becomes rational. Additionally, we established the reference intervals for the CBC parameters, and indicated the necessity of gender-specific analysis for them.

However, some limitations of present study should not be neglected. First, the reference intervals for CBC parameters is susceptible to the region of the participant.^[11]^ And our study was conducted in a single region. Therefore, our results could not represent the real reference value of adults aged 80 to 89 years in China, even other countries. Secondly, some of the other CBC parameters, such as reticulocyte-associated parameters and nucleated RBC were not showed in present research due to insufficient availability of facilities. These parameters also contribute to the diagnosis of disease. For example, previous studies have shown that population reticulocyte levels were inversely associated with atherosclerosis,^[45]^ and nucleated RBC count was associated with significantly elevated mortality risk of neonate.^[46]^ Because of the above shortcomings, further studies should focus on the reference intervals in elderly individuals.

## 5. Conclusions

The reference intervals of CBC parameters were established for individuals aged 80 to 89 years in Guizhou, China. Some of our results were sex-specific, and most of our results show lower values while comparing with commonly used reference intervals in China. Thus, more attentions should be paid to these differences, and there is need to establish region-specific reference intervals of CBC for elderly population. More research is needed to determine the reference intervals of CBC for healthy elderly population in China, which would be helpful to clinical diagnosis and decisions-making.

## Acknowledgments

We are grateful to volunteers for providing their clinical information.

## Author contributions

**Conceptualization:** Xun Min, Jinlie Long, Xiuhong Wang, Yuanzhong Zhou.

**Data curation:** Jinlie Long, Xiuhong Wang.

**Formal analysis:** Jinlie Long, Xiuhong Wang.

**Project administration:** Xiuhong Wang, Jianbo Yuan, Jianru Yang, Yuan Chen.

**Software:** Jinlie Long, Jie Zhou, Enxi Hu.

**Supervision:** Xun Min, Yuanzhong Zhou.

**Writing – original draft:** Jinlie Long.

**Writing – review & editing:** Xun Min, Jinlie Long, Yuanzhong Zhou.
